# On Pseudo-Syphilis, with Reflections on the Case of W. O.

**Published:** 1815-03

**Authors:** Richard Walker

**Affiliations:** Oxford


### *For the Medical and Physical Journal.*

### *On Pseudo-syphilis, with Reflections on the Case of W. O.;* by Mr. R. Walker.

THERE is a new disease, or rather perhaps an old affection with a new name, introduced of late years to the attention of the faculty, denominated Pseudo-syphilis, which, it is said, is so like true syphilis in its appearance and symptoms as to be occasionally mistaken for it.

It is asserted by a professional gentleman of great celebrity, first, that the appearances and symptoms* of pseudo-syphilis, which he has thus named, are so exactly similar to true syphilis, that even he himself has not been able to ascertain or discriminate, from inspection alone, the difference between the two, and that he is obliged to resort to other means for this purpose; secondly, that he has acquired the means, however, from the history of the case;—the irregular progress of the disease (true syphilis uniformly getting worse, if its progress be not arrested or prevented by mercury), but more especially by the more rapid progress of the constitutional symptoms, as sore throat, eruptions, &c. and the more quick, sudden, and irregular changes, from one symptom or affection to another, than in true syphilis; and, thirdly, in consequence of mercury, in any manner exhibited, having no good effect upon them, but otherwise; which is never the case, by a judicious management of mercury in true syphilis, and therefore requiring very different means.

Even the primary sores, though ordinarily wanting the distinctive character of chancre, are occasionally so like as not to be distinguished from that.

This disease, or affection, is supposed to originate, or to be produced by the absorption of morbific animal matter into the system of a nature different from that which causes true syphilitic affections.

Moreover, it is asserted, that this may happen without sexual intercourse; and even be absorbed into the habit without a previous sore.

It is the commonly received opinion, at this time, I believe, that the matter, or discharge, which is produced in gonorrhœa virulenta is of a different specific nature from that of the syphilitic virus; (I remember the time, however,

* Viz. the syphilitic sore throat, eruptions, nodes, and in short every symptom of constitutional affection which is incident to true or genuine syphilis.

when it was considered the same in both instances) and that the latter can be arrested in its progress, or cured, only by mercury; whereas, it is well known, that gonorrhœa virulenta is cured without mercury; likewise, that, if left alone, it occasionally gets well, or exhausts itself, without the application of any remedy; and, moreover, however neglected or ill-treated, produces no constitutional effect, or secondary symptoms depending upon absorption of the virus into the system.

I believe, however, the fact respecting the different specific nature of the two has never yet been clearly and satisfactorily demonstrated; and, likewise, that the advocates of the former opinion furnish, as they think, satisfactory reasons of the identity of the two, viz. as principally depending upon the virulent matter being received either upon a secreting or non-secretic surface, as productive either of gonorrhœa virulenta, or chancre, and its consequent effects; the secreting surface being favourable for the former of these affections, and the non-secreting surface for the latter. Upon the whole, however, it must be admitted, according to ordinary facts, that there are greater difficulties in the way of the formerly received opinion than the present; moreover, the well known fact, that gonorrhœa virulenta, and all its consequent affections, can be cured without mercury; and the syphilitic affection, viz. chancre and its consequences, cannot, is sufficient as a rule for practice between them.

Since, however, it is probable, that every specific animal poison is capable of producing certain constitutional and secondary effects upon the human system, according to its peculiar nature, as in the instances of bites and stings from venomous animals, the absorption of putrid animal matter, &c. may we not, admitting the singular and newly-discovered fact respecting those affections which resemble syphilis so nearly as to have obtained the name of pseudo-syphilis, that this newly-distinguished affection may be derived and produced by the absorption of the matter or virus of gonorrhœa virulenta, considering this as a different modification of syphilis, or venereal affection, only, especially since it is ascertained that the secondary affection of this newly-described disease is, like gonorrhœa virulenta, itself curable without mercury, and likewise, as it is said, occasionally goes off again, or disappears by exhausting itself, without the assistance of medicine.

It is true, the discoverers of a new affection, or the propagators of a new doctrine, are too often led away, or too apt to indulge in certain fanciful ideas respecting it; and, I must confess, that in the present instance, certain observations tions and opinions advanced, reminded me of a work, entitled *Lucina sine Concubitu*, and hence, I apprehend, some persons may have been led to treat the whole as not deserving of serious regard, as appears, I think, by occasional ludicrous allusions, as I have considered them, to this subject.

If it be ascertained, however, that, in this country, there is a false or rather spurious species of syphilis arising from sexual intercourse, produced by absorption of a different species of virus into the system, and requiring a different mode of treatment, its characteristic difference from true or genuine syphilis should be generally known, otherwise, I apprehend, especially amongst the younger part of the profession, very serious consequences may arise from the mere promulgation of such a fact, without the precise means of discrimination.

I have been led to these observations, as well from the knowledge of cases actually of a syphilitic nature, which were cured by mercury, which had been supposed to be, as I presume, of a pseudo-syphilitic nature, having been ineffectively treated without mercury before; as from a conviction that I may have erred occasionally myself on the other side. All circumstances considered, however, I am of opinion, that an error on the first side of the question is the greater evil of the two.

It is, perhaps, scarcely necessary to observe, that, although the internal exhibition of mercury alone might, judiciously managed and duly persisted in, effect a cure in most or all syphilitic affections, yet the only perfectly effective means is by mercurial inunction, which acts as specifically, as it were, upon this disease as water upon fire.

Moreover, I consider the mode of cure, by the internal exhibition of mercury alone, excepting where, from any cause, mercurial inunction cannot be complied with, as an unnecessary and injurious annoyance of the stomach and bowels, especially the more acrid preparations of mercury, as the corrosive sublimate.

It must not be forgotten, however, that, although the internal absorption of mercury into the system is, ordinarily speaking, a certain specific or antidote in syphilitic affections, yet the occasional application of topical means, not of a mercurial nature, are essential in certain local affections, as chancrous sores, ulcerated bubo, &c. and which, by the bye, forms an intricate as well as essential part of the treatment;* and,

* Ill-conditioned eroding chancrous sores and spreading ulcerated buboes, were once formidable difficulties to me, especially among and, likewise, to know when and where to stop the exhibition of mercury; the latter circumstance may ordinarily be known by the sores changing to a kind of putrid tendency.

I have not unfrequently met with instances of persons who have come to me with a supposed venereal affection, which, on examination, I found to be an ordinary sore, or even a mere abrasion of the part, arising probably from friction. In these instances, I judged it prudent to administer a mild mercurial, viz. the Pilulæ hydrargyri, leaving the sore to itself, or dressing it with an application of no specific character.

Such sores quickly healed, the patient had not been annoyed by the mercurial; and both the patient and myself remained satisfied.

The same line of conduct I should be inclined to pursue in all dubious cases, being well aware that by such means I might detect the presence of such affections as were of a true syphilitic nature, or, with impunity to the patient, discover such as were not so.

The most perplexing kind of cases I have ordinarily met with, both respecting a true discrimination and the fittest mode of treatment, is, where syphilis has been grafted, as it were, upon scrophula. It is extremely difficult, or rather almost impossible, to persons in ordinary practice, to believe that certain cases of an assumed pseudo-syphilitic nature, so closely resembling the true syphilis, as they are described to be, can be any other than the disease itself, rendered obscure, perhaps, by being combined with some other constitutional affection, or a different modification, as hinted at above, of a venereal affection. In either case, a cautious administration of mercury, by way of trial, by means of the Pilulæ hydrargyri, or mercurial frictions, or, according to circumstances, both conjointly, which I consider to be the most effective means, cannot be productive of any evil consequences, and must necessarily point out the true line of practice to be pursued. I am strongly of opinion, if the gentleman who described his case himself, provided it be a correct relation of it, in the Medical and Physical Journal for December last, had adopted this plan at first, instead of taking the corrosive sublimate, he would soon have amended, and, by perseverance, been cured.

---

among persons compelled to pursue their ordinary avocations, notwithstanding the best information I could obtain from men and books; but further experience and attention has enabled me to reduce the treatment of them to a kind of mechanical command or certainty.

I shall

I shall take this opportunity of noticing a circumstance which has recently occurred, although perfectly irrelevant to the present subject.

An artificer applied to me, having some time before received an injury in the middle joint of the fore-finger of the right hand, but which he had not before considered to be of a serious nature.

The joint was inflamed and enlarged with a small wound evidently communicating with the joint. The finger continued to increase in size and inflammation, to an intense degree, but there was no fluctuation or apparent collection of matter.

At length, a thin glairy fluid, mixed with pus, issued from the wound, in the course of which, the tumor and inflammation gradually subsided, and the bones at the joint grated and became quite loose; the remaining tumor and inflammation, at length, entirely disappeared, and in the end, about six weeks from the time the injury was received, the wound was healed, and the joint perfectly consolidated, viz. a fixed joint with a certain degree of flexure in it.

During the inflammatory state of the finger, a linseed poultice was applied; when this had completely or nearly subsided, a dressing with simple cerate was applied, with splints and roller, tightening the roller by degrees as the finger would bear it.

Having once ascertained the state of the joint, by an accurate examination, I forbore every thing of this kind afterwards, keeping the part as free from motion as possible, taking particular care, at each time of dressing, to press, or milk out as it were, the matter at the wound from above and below the joint; from the intensity of the swelling and inflammation a large portion of the cuticle separated of course and was removed.

In the course, or progress, of this case, a professional gentleman of eminence was applied to, whose opinion was decidedly, that the finger could not be preserved, which most probably might have been the same with most others of the profession.

It appeared to be important to the patient, notwithstanding the stiffness of the joint, of which I apprised him, for the finger to be preserved; care was taken, however, in the course of the cure, to favour, as much as possible, a due degree of flexed state in the joint.

I mention this case to show the fair prospect there is of preserving limbs, when the injury in the joint arises, not from a constitutional taint, as in scrophula, &c. but from incidental incidental injury, and constantly to bear that distinction in mind.

By the same mode of treatment, I have succeeded in the larger joints, as the knee, or elbow-joint, even in some, after they have been condemned to amputation, and in some instances, the same mode has succeeded even in scrophulous joints by very long perseverance.

Care should be taken, in the course of the cure, that a due degree of straitness, or flexure, be preserved, according as the nature and use of the limb may require, which is promoted much by a due form and application of the splints, viz. either strait or flexed.

In the smaller joints, as the finger, the splints are best made of thin metal, as tin, with a due degree of flexure, and applied to each side of the joint, confined by a roller.

The finger was never, till nearly well, dressed less than twice in the day, and, whilst at worst, thrice in the twenty-four hours, taking especial care to press out the matter each time, after the manner described, upon which latter circumstance, much of the success in such cases depends. In the course of the cure, it became necessary once or twice, in consequence of the wound prematurely closing, to puncture it with a lancet, and to use a stimulating application under the cerate to keep it open.

*Oxford; Jan.* 1815. RICHARD WALKER.

